# *Lactobacillus plantarum* HY7715 Ameliorates Sarcopenia by Improving Skeletal Muscle Mass and Function in Aged Balb/c Mice

**DOI:** 10.3390/ijms221810023

**Published:** 2021-09-16

**Authors:** Kippeum Lee, Jisoo Kim, Soo-Dong Park, Jae-Jung Shim, Jung-Lyoul Lee

**Affiliations:** R&BD Center, hy Co., Ltd., 22, Giheungdanji-ro 24beon-gil, Giheung-gu, Yongin-si 17086, Korea; joy4917@hanmail.net (K.L.); jkim136@hy.co.kr (J.K.); soodpark@hy.co.kr (S.-D.P.); jjshim@hy.co.kr (J.-J.S.)

**Keywords:** aging, probiotics, *Lactobacillus plantarum* HY7715, sarcopenia, skeletal muscle

## Abstract

Sarcopenia is a loss of muscle mass and function in elderly people and can lead to physical frailty and fall-related injuries. Sarcopenia is an inevitable event of the aging process that substantially impacts a person’s quality of life. Recent studies to improve muscle function through the intake of various functional food materials are attracting attention. However, it is not yet known whether probiotics can improve muscle mass and muscle strength and affect physical performance. *Lactobacillus plantarum* HY7715 (HY7715) is a lactic acid bacteria isolated from kimchi. The present research shows that *L. plantarum* HY7715 increases physical performance and skeletal muscle mass in 80-week-old aged Balb/c male mice. HY7715 not only induces myoblast differentiation and mitochondrial biogenesis but also inhibits the sarcopenic process in skeletal muscle. In addition, HY7715 recovers the microbiome composition and beta-diversity shift. Therefore, HY7715 has promise as a functional probiotic supplement to improve the degeneration of muscle function that is associated with aging.

## 1. Introduction

Skeletal muscle is one of the largest organ in the body, accounting for about 40–50% of the body mass [1,2]. The preservation of skeletal muscle function is important to maintain whole-body energy homeostasis and the capacity to perform activities associated with daily living. Skeletal muscle has a high regenerative ability; moreover, cellular and molecular signaling pathways within skeletal muscle promote myoblast activation, proliferation, and differentiation [3]. Skeletal muscle development is a complex process that is controlled through numerous complementary interactions that balance protein synthesis and protein degradation [4,5]. A balance between muscle synthesis and degradation is important to maintain appropriate muscle mass, thus preventing hypertrophy and atrophy [6].

During aging, there is an age-induced loss in skeletal muscle mass and function, known as sarcopenia. Sarcopenia is associated with biological, structural, molecular, and functional changes in skeletal muscle. These changes lead to decreased mobility and increased susceptibility to falls, various diseases, and mortality [7,8]. As society ages, the occurrence of physical limitations increases rapidly, reducing human quality of life and increasing medical costs [9]. Therefore, it is important to study ways in which muscle tissue can be developed and loss of muscle mass can be prevented, for example, through nutritional intake. Moreover, recent in vitro and in vivo studies suggest that modulating the molecular and cellular changes that occur during skeletal muscle aging is a promising antisarcopenia strategy [10,11,12].

Myoblast differentiation and the growth of myogenic cells are orchestrated by the expression of myogenic determination protein (MyoD), and myosin heavy-chain 1 (MYH1). Briefly, sequential upregulation of MyoD and MYH1 expression is essential to develop muscle fibers [13,14,15]. In particular, the terminal differentiation mediator, MYH1, leads to the production of muscle filaments. Several studies have investigated the cellular and molecular changes that occur during the skeletal muscle loss and dysfunction that is associated with sarcopenia. Degradation of muscle protein involves components of the ubiquitin–proteasome pathway, including E3 ubiquitin ligases, such as myostatin, F-box protein (Atrogin1), and muscle RING-finger 1 (MuRF1) [16,17]. In addition, muscle expresses mitochondrial-related genes, including *TFAM1*, *PGC1α*, and *UCP3*, which regulate energy metabolism and prevent physiological fatigue [18,19]. TFAM1 and PGC1α are transcription factors associated with sarcopenia and metabolic disease during aging. UCP3 is primarily found in skeletal muscle and has a vital role in regulating mitochondrial biogenesis and energy expenditure in skeletal muscle [20,21]. Numerous studies have shown that the accumulation of damaged mitochondria causes myofiber death, suggesting an association with sarcopenia. Since normal expression of these genes is important for conserving mitochondria and maintaining cellular homeostasis, their deletion can lead to muscle degeneration [22,23].

Probiotics are live microbiota that provide health benefits and include various strains of *Lactobacillus, Bifidobacterium*, and *Saccharomyces* [24]. In particular, *Lactobacillus plantarum (L. plantarum)* is a Gram-positive, lactic acid bacteria found in many types of food including dairy, fish, and fermented vegetable foods. Recent studies have shown that *L. plantarum* has various biological actions such as anti-oxidative, antibacterial, anti-obesity, and anticancer effects, as well as metabolic-regulating activities and beneficial effects on gut inflammation and intestinal health [25,26,27,28,29]. However, the effect of *L. plantarum* on physical performance, including muscle mass and strength, has not yet been evaluated. *Lactobacillus plantarum* HY7715 (HY7715) is a probiotic patented in the Korean Collection for Type Cultures (KCTC 13101BP), isolated from kimchi, and is a unique lactic acid bacterium that is resistant to acids, including bile acid. In this study, we aimed to evaluate the effects of HY7715 on muscle myogenesis and growth in 80-week-old, aged Balb/c mice and the C2C12 cell line.

## 2. Results

### 2.1. L. plantarum HY7715 Increases Skeletal Muscle Mass in 80-Week-Old Mice

**Figure 1:**
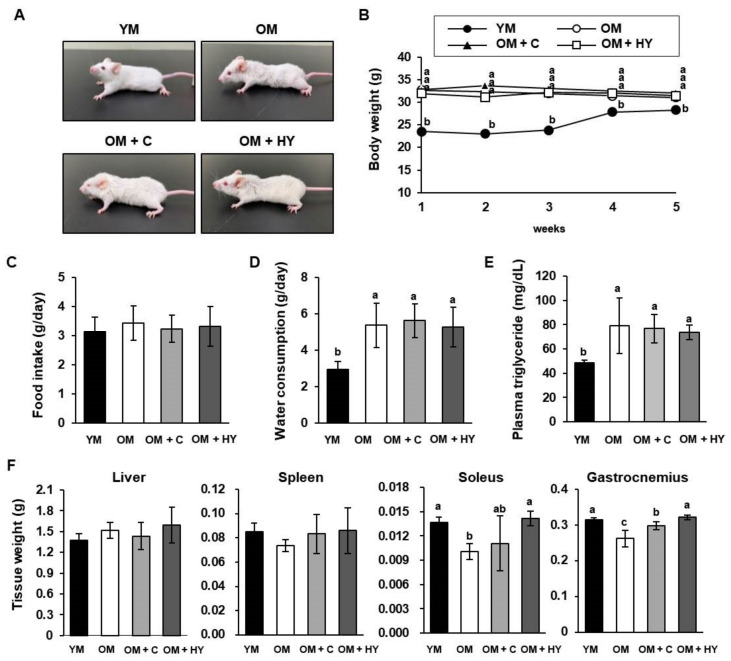
The effect of HY7715 treatment on age-induced muscle loss in mice. (**A**) Representative photographs of the mouse groups after 5 weeks of treatment. (**B**) Weekly body weight measurement, (**C**) food intake, and (**D**) water consumption per day. (**E**) Plasma triglyceride concentrations were detected using a commercial colorimetric enzyme-linked immunosorbent assay (ELISA) kit. (**F**) Tissue weights of liver, spleen, soleus muscle, and gastrocnemius muscle were measured. Statistical significance was determined using one-way ANOVA followed by Tukey’s post hoc test (N = 6). Datasets denoted by different letters are significantly different; *p* < 0.05 (a > b > c). YM, young mice; OM, old mice; OM + C, creatine-treated old mice; OM + HY, HY7715-treated old mice.

To evaluate the effects of HY7715 in vivo, we used young mice (YM, 8-weeks-old) and old mice (OM, 80-weeks-old). OM received orally administered 1 × 10^8^ CFU/kg/day of HY7715 (OM + HY) or 75 mg/kg/day of creatine (OM + C; used as a positive control) for 5 weeks. During the experimental period, the bodyweight of OM was higher than that of YM, and there was no significant difference in the body weight of the HY7715- or creatine-treated OM groups (Figure 1A,B). There was no statistically significant difference in food intake between the groups, but OM consumed more water than YM (Figure 1C,D). As shown in Figure 1E, the plasma triglyceride concentration of the OM group treated with HY7715 was 48.3 mg/dL, whereas the plasma triglyceride concentration in the control groups was 79.0 mg/dL (OM), 73.7 mg/dL (OM + C), and 76.7 mg/dL (OM + HY). Next, we measured the tissue weight of the liver, spleen, soleus muscle, and gastrocnemius muscle. As shown in Figure 1F, HY7715 and creatine did not change the phenotypic weight of the liver and the spleen. However, the weight of the soleus muscle and the gastrocnemius muscle in untreated OM was markedly reduced, suggesting that the muscle mass was decreased due to natural aging. HY7715 administration prevented this reduction and was more effective than creatine in preventing muscle loss. 

### 2.2. L. plantarum HY7715 Enhances Muscle Strength in 80-Week-Old Mice

**Figure 2:**
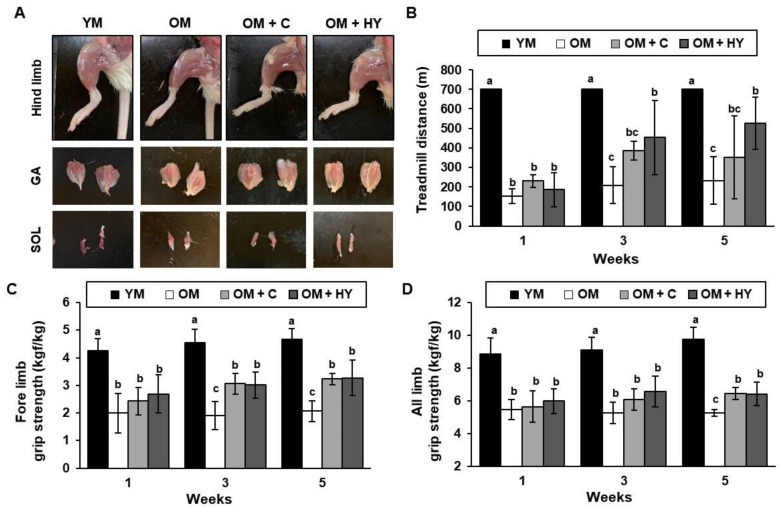
The effect of HY7715 treatment on skeletal muscle strength in aged mice. (**A**) Representative photographs of hind limb muscle (**top**), gastrocnemius muscle (**middle**), and soleus muscle (**bottom**) in the mouse groups after 5 weeks of treatment. (**B**) The running endurance of the mice was evaluated by a treadmill test. The grip strength of the forelimb (**C**) and all limbs (**D**) was measured at 1, 3, and 5 weeks and calculated per body weight. Statistical significance was determined using one-way ANOVA followed by Tukey’s post hoc test (N = 6). Datasets denoted by different letters are significantly different; *p* < 0.05 (a > b > c). YM, young mice; OM, old mice; OM + C, creatine-treated old mice; OM + HY, HY7715-treated old mice. GA, gastrocnemius; SOL, soleus.

Skeletal muscle is the most abundant and regenerative organ in the mammalian body but can be functionally compromised due to aging. Loss of skeletal muscle mass can cause physical dysfunction and imbalance. In particular, age-related loss in skeletal muscle mass and quality increases the risk of sarcopenia. In this study, we determined the impact of aging and HY7715 on the formation of hindlimb muscle and muscle strength (Figure 2A). A treadmill exhaustion test and a grip strength test were used to determine the effect of HY7715 on exercise capacity in aged mice. We used low-intensity running up to 25 m/min to test and record the exhausted time in mice. As anticipated, the treadmill distance of OM was dramatically shorter than that of YM throughout the experiment (Figure 2B). The treadmill distance of the OM + HY group was significantly longer than that of the OM group and was slightly longer than that of OM + C at weeks 3 and 5. In the forelimb and all-limb grip tests, HY7715-treated OM had significantly better maximal muscle strength than untreated OM at weeks 3 and 5 (Figure 2C,D). Of note, HY7715 significantly increased the relative forelimb (by 1.58-fold) and all-limb (by 1.22-fold) grip strength compared with the untreated OM group. 

### 2.3. L. plantarum HY7715 Improves Physiological Fatigue in 80-Week-Old Mice

**Figure 3:**
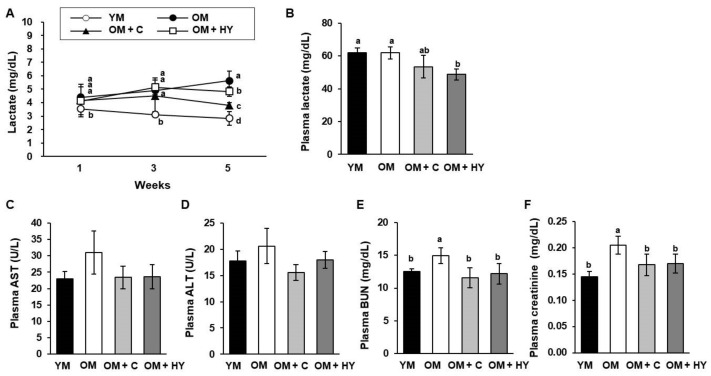
The effect of HY7715 treatment on physiological fatigue in aged mice. (**A**) Blood was sampled from the tail vein after the treadmill test, and lactate was analyzed using a lactate sensor. After sacrifice, (**B**) plasma lactate, (**C**) plasma aspartate aminotransferase (AST), (**D**) plasma alanine aminotransferase (ALT), (**E**) plasma blood urea nitrogen (BUN), and (**F**) plasma creatinine concentrations were detected using a commercial colorimetric enzyme-linked immunosorbent assay (ELISA) kit. Statistical significance was determined using one-way ANOVA followed by Tukey’s post hoc test (N = 5). Datasets denoted by different letters are significantly different; *p* < 0.05 (a > b > c > d). YM, young mice; OM, old mice; OM + C, creatine-treated old mice; OM + HY, HY7715-treated old mice.

Skeletal muscle fatigue can be stimulated after intense exercise, resulting in a decreased ability to produce physical force [30]. Exercise-induced muscle fatigue can be evaluated by measuring biochemical markers, including lactate, AST, ALT, BUN, and creatine levels [31]. We measured the blood lactate level obtained from the tail vein immediately after treadmill exercise at 2-week intervals and measured the plasma lactate concentration after final dissection. At week 3, the blood lactate concentration was significantly lower in YM than in OM groups; at week 5, there was a significant difference between the OM groups (OM, OM + C, OM + HY). The post-exercise lactate concentration was lowest in HY7715-treated OM (Figure 3A). Although the plasma lactate concentration was lowest in the OM + HY group, it appears that both YM and OM maintain some degree of lactate homeostasis. (Figure 3B). The plasma AST and ALT levels of OM + HY were not significantly different compared with those in untreated OM but were 0.76-fold and 0.87-fold lower, respectively (Figure 3C,D). The levels of BUN and creatinine, which are synthesized in muscle cells and secreted into the blood, are indicators of age-related muscle damage. As shown in Figure 3E,F, the BUN and creatinine levels were significantly higher in untreated OM than in YM; treatment with HY7715 or creatine reduced BUN and creatinine levels close to those observed in the YM group. 

### 2.4. L. plantarum HY7715 Promotes Muscle Development in 80-Week-Old Mice

**Figure 4:**
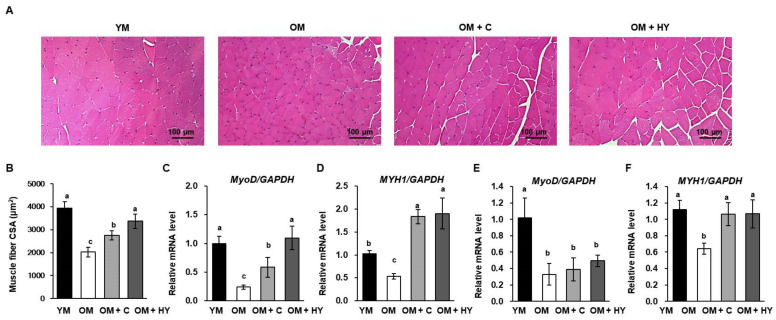
The effect of HY7715 treatment on myogenic development and differentiation in aged mice. (**A**) Hematoxylin and eosin (H&E) staining of limb muscle fiber after 5 weeks of treatment. (**B**) The cross-sectional area (CSA) of muscle fibers were quantified. The mRNA level of *MyoD* and *MHY1* in gastrocnemius muscle (**C**,**D**) and soleus muscle (**E**,**F**) were normalized to the level of *GAPDH* mRNA and calculated as a relative-fold value. Statistical significance was determined using one-way ANOVA followed by Tukey’s post hoc test (N = 5). Datasets denoted by different letters are significantly different; *p* < 0.05 (a > b > c). YM, young mice; OM, old mice; OM + C, creatine-treated old mice; OM + HY, HY7715-treated old mice.

We investigated the effect of HY7715 on skeletal muscle development in aged mice. Histological analysis of the gastrocnemius muscle in the hindlimb (Figure 4A,B) showed that HY7715 treatment increased the cross-sectional area of the gastrocnemius muscle in OM, and this increase was greater than that produced by creatine. The fundamental mediators for myogenesis, MyoD and MYH1, are necessary for muscle development and the early response to muscle damage [32]. The change in hindlimb muscle expression of MyoD might be associated with a lower expression of myogenic differentiation markers in aged mice. As shown as Figure 4C,D, mRNA levels of MyoD and MYH1 in the gastrocnemius muscle were lower (24% and 33%, respectively) in OM than in YM; oral administration of HY7715 to OM increased the levels of MyoD and MYH1 by 61% and 49%, respectively, of those of observed in YM and was more effective than creatine. In soleus muscle, HY7715 treatment slightly increased MyoD levels; MyoD expression was higher in HY7715-treated mice than in uncreated OM. The expression of MYH1 in mice treated with HY7715 was similar to that in YM (Figure 4E,F).

### 2.5. L. plantarum HY7715 Ameliorates Muscle Atrophy Regulators in 80-Week-Old Mice

**Figure 5:**
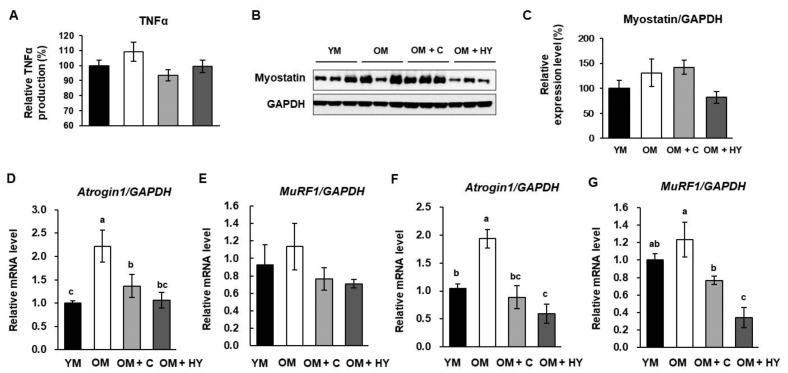
The effect of HY7715 treatment on age-induced sarcopenic factors in mice. (**A**) The plasma level of TNFα in mice was measured using a commercial colorimetric enzyme-linked immunosorbent assay (ELISA) kit. (**B**) Western blot analysis of myostatin and glyceraldehyde 3-phosphate dehydrogenase (*GAPDH*) in gastrocnemius muscle of the mice. The mRNA levels of muscle atrophy F-box gene (*Atrogin1*) and muscle RING-finger protein 1 (*MuRf1*) in gastrocnemius muscle. (**C**) The protein level of myostatin was quantified. The mRNA level of *Atrogin1* and *MuRf1* in gastrocnemius muscle (**D**,**E**) and soleus muscle (**F**,**G**) were normalized to the level of GAPDH mRNA and calculated as a relative-fold value. Statistical significance was determined using one-way ANOVA followed by Tukey’s post hoc test (N = 5). Datasets denoted by different letters are significantly different; *p* < 0.05 (a > b > c). YM, young mice; OM, old mice; OM + C, creatine-treated old mice; OM + HY, HY7715-treated old mice.

TNFα is an inflammatory cytokine that can induce muscle atrophy and muscle degradation [17]. Increased TNFα expression in OM mice paralleled skeletal muscle atrophy and correlated with increasing levels of Atrogin-1 and MuRF1. As shown in Figure 5A, aging increased TNFα production and HY7715 reduced TNFα production in OM. Skeletal muscle loss can occur due to increased protein breakdown, which is mediated by the E3 ubiquitin ligases including myostatin, Atrogin1, and MuRF1 [17]. The protein expression levels of myostatin were higher in the untreated OM group than in YM, and HY7715 significantly reduced the protein level of these ligases (Figure 5B,C). In addition, the mRNA level of *Atrogin1* and *MuRF1* in both the gastrocnemius and soleus muscle in untreated OM was higher, respectively, than in YM. However, HY7715 downregulated the mRNA level of *Atrogin1* and *MuRF1* in the gastrocnemius and soleus muscle, respectively (Figure 5D–G). These results suggest that HY7715 prevents sarcopenia by inhibiting age-induced muscle atrophy. 

### 2.6. L. plantarum HY7715 Upregulates Mitochondrial Biogenesis Factors in 80-Week-Old Mice

**Figure 6:**
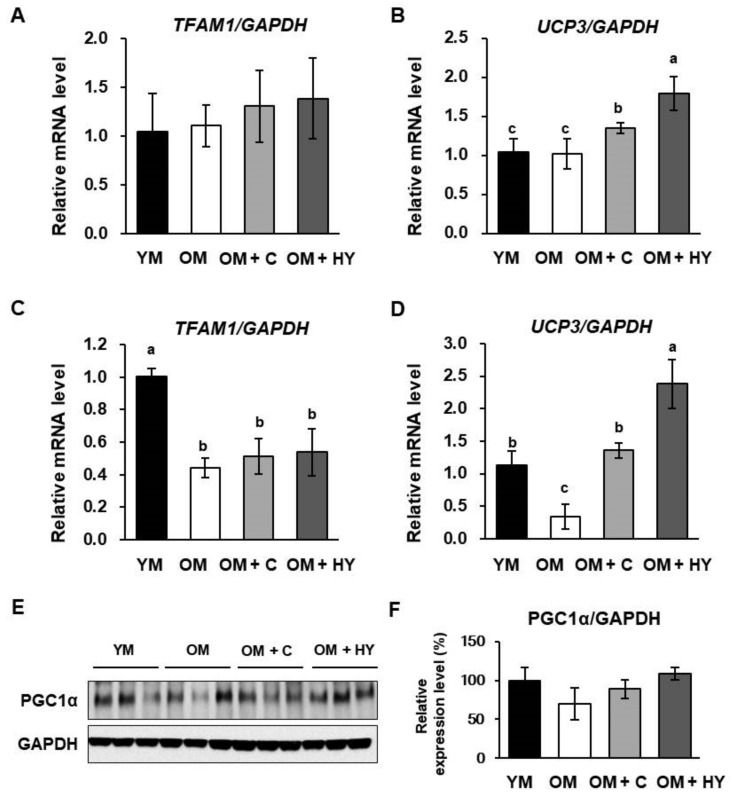
The effect of HY7715 treatment on energy metabolism factors in mice. (**A**) The mRNA level of transcription factor A, mitochondrial (*TFAM1*), and uncoupling protein 3 (*UCP3*) in gastrocnemius muscle (**A**,**B**) and soleus muscle (**C**,**D**) were normalized to the level of glyceraldehyde 3-phosphate dehydrogenase (*GAPDH*) mRNA and calculated as a relative-fold value. (**E**) Western blot analysis of peroxisome proliferator-activated receptor gamma coactivator 1-alpha (PGC1α) and GAPDH in gastrocnemius muscle from the mice. (**F**) The protein level of PGC1α was quantified. Statistical significance was determined using one-way ANOVA followed by Tukey’s post hoc test (N = 5). Datasets denoted by different letters are significantly different; *p* < 0.05 (a > b > c). YM, young mice; OM, old mice; OM + C, creatine-treated old mice; OM + HY, HY7715-treated old mice.

Aging may affect mitochondrial function, in particular the regulation of the expression of genes that regulate mitochondrial biosynthesis in skeletal muscle. This finding suggests that preventing mitochondrial changes could be an effective therapy for sarcopenia. We determined the impact of aging and HY7715 treatment on the expression level of *TFAM1* and *UCP3* in muscle. As shown in Figure 6A,B, there was no significant difference in *TFAM1* and *UCP3* expression between YM and untreated OM, but *TFAM1* and *UCP3* expression were higher in the soleus muscle of the OM + HY group than in the other groups. In addition, HY7715 did not significantly increase the mRNA levels of TFAM1 in gastrocnemius muscle, but increased the level of UCP3 expression such that it was higher than that in YM (Figure 6C,D). Furthermore, HY7715 treatment increased the protein expression level of PGC1α in the gastrocnemius muscle (Figure 6E,F).

### 2.7. L. plantarum HY7715 Recovers the Microbiome Composition and Beta-Diversity Shift in 80-Week-Old Mice

**Figure 7:**
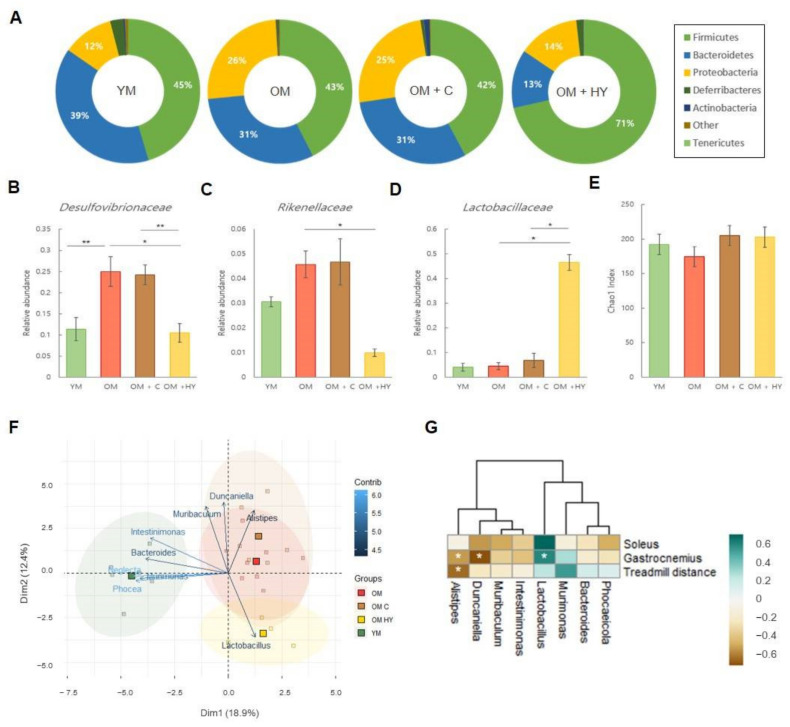
The effect of HY7715 treatment on the abundance of bacterial taxa in mouse cecum. (**A**) The relative abundance of phyla in each treatment group. (**B**–**D**) Family-level taxa of the most abundant bacteria. (**E**) The α-diversity of each group (Chao1 index). (**F**) A plot of the principal component analysis (PCA) scores showing the variation of each treatment group and the contribution of bacteria at the genus level. Dimension 1 (Dim1) explains 18.9% of the variance and dimension 2 (Dim2) explains 12.4% of variance. (**G**) Spearman’s correlation analysis was performed between the bacterial genus-level taxa and the measured physiological factors (muscle mass and treadmill distance). YM, young mice; OM, old mice; OM + C, creatine-treated old mice; OM + HY, HY7715-treated old mice; contrib; contribution. N = 5, The Mann–Whitney U test was used for statistical analysis. Data are expressed as the mean ± SEM (* *p* < 0.05 and ** *p* < 0.01).

We analyzed the difference in composition of gut microbes of each group of mice and observed differences in the specific microbial taxa. As shown in Figure 7A, Firmicutes and Bacteroidetes comprised 84.6% of the gut microbiome in YM and 84.5% of the gut microbiome in the OM + HY group. However, Firmicutes and Bacteroidetes were lower in the OM and OM + C groups; Firmicutes and Bacteroidetes comprised 73.5% of the gut microbiome in the OM group and 72.7% of the gut microbiome in the OM + C group. The proportion of Proteobacteria were as follows: 11.4% in the YM group, 13.8% in the OM + HY group, 25.6% in the OM group, and 25.1% in the OM + C group. Next, we analyzed the relative abundance of gut microbiota at the taxonomic level in each group of mice (Figure 7B–D). At the family level, Desulfovibrionaceae were higher in the OM and OM + C groups than in YM (*p* = 0.00649 and *p* = 0.0116, respectively). The level of Rikenellaceae was significantly lower in YM than in the OM group (*p* = 0.0201) and Lactobacillaceae were significantly higher in the OM + HY group than in untreated OM and in the OM + C group (*p* < 0.0001 and *p* < 0.0001, respectively). To evaluate the gut microbial diversity, the Chao1 index and PCA were measured. The Chao1 index (Figure 7E) showed no significant difference in the α-diversity of the intestinal microbial community between the groups. However, the scatter plots of PCA showed that some groups were separated from other groups (Figure 7F). At a genus level, *Alistipes*, *Duncaniella*, *Muribaculum*, and *Lactobacillus* contributed the most in dimension 2 (12.4%), and *Neglecta*, *Phocea*, *Murimonas*, *Bacteroides*, and *Intestinimonas* showed an important variation in dimension 1 (18.9%). Our data indicated that PCA based on genus-level bacterial composition was different in YM than in untreated OM, the OM + C group, and the OM + HY group. *Lactobacillus* was closely associated with the OM + HY group; while *Alistipes* and *Duncaniella* were associated with the untreated OM and the OM + C groups (Figure 7F). Lastly, we analyzed Spearman correlations of muscle phenotypes (such as soleus weight, gastrocnemius weight, and treadmill distance) between the genus taxonomic levels that contributed the most in PCA. *Lactobacillus* positively correlated with soleus and gastrocnemius weight, whereas *Duncaniella* and *Alistipes* negatively correlated with soleus and gastrocnemius weight (Figure 7G). *Alistipes* negatively correlated with gastrocnemius weight and treadmill distance (Figure 7G).

### 2.8. L. plantarum HY7715 Impacts Myogenic Activation in C2C12 Cells

**Figure 8:**
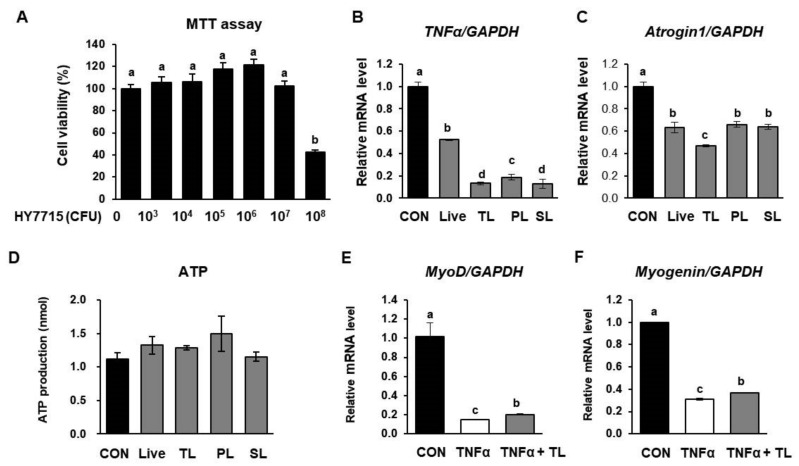
The effect of different cell fractions of HY7715 on C2C12 myoblast differentiation. (**A**) An MTT assay in the C2C12 cell line. The mRNA level of (**B**) tumor necrosis factor-α (*TNFα*) and (**C**) muscle atrophy F-box gene (*Atrogin1*) in C2C12 cells treated with HY7715 cell fractions were normalized to the level of *GAPDH* mRNA and calculated as a relative-fold value. (**D**) Adenosine triphosphate (ATP) production was detected using a commercial colorimetric enzyme-linked immunosorbent assay (ELISA) kit. The mRNA level of (**E**) myoblast determination protein 1 (*MyoD*) and (**F**) *myogenin* in C2C12 cells treated with HY7715 cell fractions were normalized to the level of *GAPDH* mRNA and calculated as a relative-fold value. Statistical significance was determined using one-way ANOVA followed by Tukey’s post hoc test (N = 5). Datasets denoted by different letters are significantly different; *p* < 0.05 (a > b > c > d). CON, control cells; Live: live whole cells of HY7715; TL, total lysates of heat-killed HY7715; PL, pellet of lysates of heat-killed HY7715; SL, supernatant of lysates of heat-killed HY7715; TNFα, 100 ng/mL TNFα-treated cells; TNFα + TL; total lysates of heat-killed HY7715 and 100 ng/mL TNFα-treated cells.

The C2C12 cell line derived from murine skeletal muscle is an established model used to investigate muscle differentiation [33,34,35]. To identify how HY7715 induces muscle myogenic activation, C2C12 cells were differentiated for 5 days, and HY7715 was added to the differentiation media for the last 48 h. Whole-cell extracts of HY7715 were lysed, and the cytoplasmic and membrane fractions were isolated. As shown in Figure 8A, we showed that HY7715 was cytotoxic at concentrations ≥10^8^ CFU/well. Therefore, we used 10^6^ CFU/well of HY7715 for subsequent experiments. The mRNA levels were compared in the total lysate, the pellet (the cell-membrane component), and the supernatant (the cytoplasmic component) obtained by centrifuging lysed HY7715 cells. The total lysate (TL) was the cell fraction that reduced the mRNA level of *TNFα* and *Atrogin1* the most in treated C2C12 (Figure 8B,C). TNFα is known to promote atrophy of muscle cells and can stimulate the expression of *Atrogin1*, a muscle degradation biomarker. We measured the ability of HY7715 to counteract the harmful effects of TNFα on differentiating myotubes by measuring muscle differentiation biomarkers. As anticipated, HY7715 regulated the expression of ATP production in myoblasts; HY7715 TL also elevated *MyoD* and *myogenin* expression in C2C12 cells treated with TNFα.

## 3. Discussion

An increasing awareness of health among the public is driving interest in functional foods and materials derived from raw material products. The effects of orally ingested microorganisms on health and the prevention of disorders have been studied for a long time, and the correlation between microbiota, health conditions, and disease development is an area of active research [36]. Probiotics are live microorganisms that provide health benefits for the host when administrated in adequate amounts. In particular, lactic acid bacteria are a major source of probiotics that are gaining popularity due to a variety of beneficial health effects. Lactic acid bacteria, including *L. plantarum,* are the major bacterial species responsible for maintaining gut health [37,38]. According to our previous study, *L. plantarum* isolated from food is safe when orally ingested and is thus suitable as a functional nutritional supplement for treating the elderly, athletes, recuperating patients, and other special or particularly sensitive populations [39]. The presence of various microbiota is related to the occurrence of disorders including gastrointestinal inflammatory disease, allergic reactions, cancer, colon disorders, obesity, and diabetes, as well as brain health [40,41,42,43]. However, few studies have reported the effect of lactic acid bacteria supplements on muscle fatigue, exercise performance, and gut microbial profile [44].

Muscle loss is a process that begins and continues from around age 30. During this process, the amount of muscle and the size of muscle fibers gradually decreases, which is the beginning of sarcopenia [45,46]. However, the qualitative and quantitative loss of muscles can be partially delayed through the intake of nutrients. Recent research has investigated the effectiveness of diet in supporting athletic performance [47]. However, the role of gut microbiota on muscle strength has rarely been elucidated. Therefore, we aimed to establish a link between gut microbiota composition and physical activity, including the notion that altering the gut microbiota composition may contribute to the physical performance of the host. We used *L. plantarum* HY7715 probiotics to test this hypothesis. To better understand the mechanism of action of HY7715 and its role in inducing muscle health benefits, we evaluated HY7715 in vitro using C2C12 myoblasts and in vivo using young and old mice. The in vivo effect of HY7715 (1 × 10^8^ CFU/kg/day) was compared with the effect of creatine (75 mg/kg/day). Creatine was used as a positive control as it is a representative dietary supplement used to enhance exercise performance that can ameliorate sarcopenia and muscle atrophy by activating direct anabolic and anticatabolic pathways [48,49].

In this present study, we showed that muscle mass and function was significantly lower in 80-week-old, aged mice than in 8-week-old mice. Skeletal muscle is constantly depleted by the fluctuating demands of its environment, and maintenance throughout an organism’s lifespan is mainly determined by the capacity of skeletal muscle to adapt and grow. Interestingly, we determined that HY7715 increased the mass of the soleus and gastrocnemius skeletal muscles without altering body weight in aged mice. The soleus is the most sensitive and important muscle that supports the hindlimb, and the gastrocnemius is the largest muscle in the hindlimb. Furthermore, our data indicate that HY7715 effectively improved the muscle strength to bodyweight ratio. Our results showed that physical endurance and grip strength increased in HY7715-treated mice to similar levels observed in creatine-treated aged mice.

Muscle fatigue is a physiological phenomenon caused by an inability to maintain the intensity of physical exercise [50]. Our data show that the skeletal muscle of elderly mice shows greater fatigue and higher lactate accumulation than that of young mice during activities that use a small amount of muscle mass. Consistent with this finding, the blood lactate level in untreated OM increased during the treadmill exercise but decreased to the basal level in HY7715-treated OM. Blood lactate is the glycolysis product of carbohydrates under anaerobic glycolysis conditions, and glycolysis is the main energy source during short-term high-intensity exercise. An increased lactate level further reduces the pH value of muscle tissues and blood, which can induce various biochemical and physiological side-effects. Therefore, blood lactate is an important blood biochemical parameter related to fatigue [51]. HY7715 also modulated plasma AST, ALT, BUN, and creatinine levels in aged mice. AST levels can be increased in a wide spectrum of clinical disorders, but an elevated ALT level is a specific indicator of tissue necrosis. Creatinine and BUN are the two major nitrogenous wastes found in blood, and our data suggest that increased muscle mass resulting from dietary intake of HY7715 contributed to changes in these muscle-fatigue factors.

Various mechanisms leading to aged-related sarcopenia have been proposed and are largely dependent on the research model used, but it is generally accepted that sarcopenia results from an imbalance between the breakdown and synthesis of muscle-fiber proteins. Age-induced loss of muscle mass is associated with decreased expression of genes involved in skeletal muscle differentiation [52,53]. The present study showed that HY7715 regulates the expression of several genes, including *MyoD* and *MYH1*, in the soleus and gastrocnemius muscles of mice. MyoD is an essential factor for terminal specification in muscle cell lineages to promote the expression of myogenic regulatory factors such as MYH1. Our analysis of the cross-sectional area of the gastrocnemius muscle fiber showed that the area was smaller in untreated OM than in YM and that HY7715 treatment increased the cross-sectional area to a similar size as the increase observed with creatine treatment. HY7715 may stimulate the myogenic activation of the gastrocnemius and soleus muscle in OM, through a mechanism involving the induction of myoblast differentiation.

Aging is associated with chronic low-grade systemic inflammation, which is known to increase circulating levels of certain cytokines such as TNFα in aged skeletal muscle. TNFα, which orchestrates cellular inflammatory and apoptotic signaling pathways, contributes to the aging process. It is reported that TNFα levels increase with age in various tissues including the liver, heart, and kidney and that elevated TNFα levels compromise the function of skeletal muscle in elderly individuals. Although the role of the ubiquitin–proteasome pathway in sarcopenia is less clear, a recent review suggested that elevated levels of pro-inflammatory mediators including TNFα might upregulate this proteolytic pathway by regulating the ubiquitin–proteasome system. These findings indicate that the catabolic activity of TNFα is closely related to muscle pathology and that pro-inflammatory cytokines are highly expressed in the patients with muscle weakness [34,54,55]. The ubiquitin E3 system is a proteolytic process required for cellular processes such as inflammatory reactions, and its importance in sarcopenia has recently been attracting attention. Myostatin, Atrogin1, and MuRF1 are expressed in skeletal muscle and inhibit cell-cycle progression and control muscle-growth factors. In this present study, HY7715 treatment significantly reduced the expression of *TNFα* and as well as that of sarcopenic factors including *myostatin*, *Atrogin1*, and *MuRF1*, which were increased in OM. Thus, our present data indicate that HY7715 may ameliorate sarcopenia by inhibiting the myoblast pro-inflammatory cytokine production and the expression of proteolytic atrophy factors.

Recent studies indicate that the composition of the gut microbiota gradually changes with age, resulting in an imbalance of the microbiome [54]. We found that aging altered the microbial composition and that aging and HY7715 administration was associated with the presence of distinct microbial taxa. Firmicutes and Bacteroidetes are two phyla that makeup around 90% of gut bacterial communities [55]. Our data show that the sum of Firmicutes and Bacteroidetes were significantly lower in OM than in YM and that the level recovered in the OM + HY group was comparable to that of the OM group. By contrast, the abundance of Proteobacteria was higher in the untreated OM group, which means that aging increased the abundance of harmful bacteria, resulting in an imbalance in the microbiota. In addition, aging increased the abundance of pro-inflammatory microorganisms, including the genus *Alistipes* belonging to the family Rikenellaceae. Our results also showed that the presence of *Alistipes* was positively associated with aging in mice, while HY7715 treatment was positively associated with the presence of *Lactobacillus*, indicating that HY7715 was stable in the intestine. We further examined the correlation between muscle mass and the relative abundance of microbial flora and found that *Lactobacillus* was positively correlated with gastrocnemius muscle mass. This finding indicates that there is a link between HY7715 administration and muscle growth by increasing myoblast differentiation and reducing the expression level of sarcopenic factors. Taken together, our results suggest that HY7715 treatment induces a healthy gut microbiome that correlates with improved muscle strength in aged animals.

In our final set of experiments, we used live-cell and heat-killed fractions of HY7715-treated cells and determined that HY7715 reduced TNFα production and the mRNA levels of sarcopenic factors during the differentiation of C2C12 cells. This effect was most pronounced in the cell lysate fraction. This result indicates that factors synthesized in the HY7715 microbiota can influence improvements in muscle function; however, it is not yet known what specific mediators are responsible for this effect, which will be investigated in further studies.

In conclusion, the current literature and the results of this study allow us to conclude that HY7715 supplementation is a potential dietary intervention to prevent sarcopenia. In addition, our findings suggest the presence of a gut–muscle axis between the gut microbiota and muscle tissues of the host, and this axis can be modulated by the administration of HY7715 probiotics. However, it is still unknown which factors in the TL fraction of HY7715 primarily act on muscle and intestinal tissue or regulate the rate of digestion and absorption; hence, it is unclear at present whether HY7715 will benefit elderly individuals. Therefore, further studies involving an elderly population need to be conducted and will form the basis of our follow-up investigation.

## 4. Materials and Methods

### 4.1. Bacterial Culture

*Lactobacillus plantarum* HY7715, originally isolated from Korean kimchi was cultured on MRS broth medium (Difco Corp., Sparks, MD, USA), and the number of colony-forming units (CFUs) were measured. *L. plantarum* HY7715 was cultured in a fermenter for 15–20 h at 37 °C, then the cells were centrifuged (8000× *g*, 4 °C) for 20 min. For animal administration, cells were freeze-dried. For in vitro studies, HY7715 cells were centrifuged at 2000× *g* for 10 min, washed twice with phosphate-buffered saline (PBS), and pellets were resuspended in PBS, pH 7.2.

### 4.2. Design of Animal Studies

Male 6-week-old and 80-week-old Balb/c mice were purchased from Doo-Yeol Biotech (Seoul, Korea). The animal studies were approved by the Institutional Animal Care and Use Committee (IACUC) of hy Co., Ltd. (IACUC approval number, AEC-2019-00012–Y). The mice were initially housed for 1 week under a 12 h light/dark cycle at 20–24 °C and 44–52% humidity to permit adaptation. After adaptation, the mice were randomly allocated to four groups (n = 7 per group) and fed for 5 weeks with AIN-93G diet (crude protein 17.9%, crude fat 7.0%, crude fiber 4.8%, moisture 7.0%, and ash 4.2%; Zeigler Bros., Inc., Gardners, PA, USA). Over the same period, HY7715 (1 × 10^8^ CFU/kg/day) or creatine (75 mg/kg/day) in an equal volume of vehicle was orally administered daily to the mice. The doses of HY7715 administered to the mice were derived from human doses (1 × 10^9^ CFU/kg/day) using a mathematical table, as previously described. The body weight, food intake, and water consumption of the mice were checked weekly. At the end of the experimental period, the mice were fasted for 12 h and euthanized using the gradual-fill method of carbon dioxide euthanasia. The tissues were collected for analysis and the organs were weighed carefully.

### 4.3. Microbiome Analysis Using Bacterial 16S rRNA Amplicon Sequencing

The bioinformatic analysis of cecal DNA samples of mice was carried out at Macrogen (Seoul, Korea). Total stool genomic DNA samples were extracted using a DNeasy PowerSoil kit (Qiagen, Hilden, Germany) according to the manufacturer’s instructions. PCR amplification of 16S rRNA sequences was conducted to prepare DNA sequencing templates. The V3 and V4 hypervariable region of the 16S rRNA gene sequence was amplified using primers targeted to the universal F/R PCR primer region according to the manufacturer’s instruction (Illumina, San Diego, CA, USA). The universal primer pairs with Illumina adapter sequences for the first set of amplifications were as follows: V3-F: 5′-TCGTCGGCAGCGTCAGATGTGTATAAGAGACAGCCTACGGGNGGCWGCAG-3′, V4-R: 5′-GTCTCGTGGGCTCGGAGATGTGTATAAGAGACAGGACTACHVGGGTATCTAATCC-3′. The PCR conditions were as follows: heat activation at 95 °C for 3 min, 25 cycles of denaturation at 95 °C for 30 s, annealing at 55 °C for 30 s, extension at 72 °C for 30 s, and a final extension at 72 °C for 5 min. PCR products were purified with AMPure beads (Agencourt Bioscience, Beverly, MA, USA) and quantified using the qPCR quantification protocol guide (KAPA library quantification kits for Illumina sequencing platforms). Paired-end sequencing was performed by Macrogen using the Illumina MiSeq platform (Illumina, San Diego, USA). After trimming [56], paired-end sequences were created using FLASH software (v. 1.2.11) [57]. The raw data were analyzed using the QIIME v. 1.9.0 program [58]. The sequencing data were filtered for low-quality reads, and mismatched indexes were trimmed. The sequences were clustered into operational taxonomic units (OTUs) with a 97% cutoff value using CD-HIT-OTU analysis [59]. After OTU clustering, the OTUs were aligned and assigned with the NCBI 16S microbial database, and taxonomy information was based on the BLAST+ database (v. 2.9.0). The Chao1 index was measured using the QIIME platform. Principal component analysis (PCA) and correlation analysis were performed and visualized using the FactoMineR package (v. 4.0.3) (available online: https://www.r-progect.org accessed on 11 December 2020) [60]. All datasets have been deposited in NCBI Gene Expression Omnibus with the accession code GSE180087.

### 4.4. Grip Strength Test

The maximal muscle strength of the mice was determined by measuring grip strength at 1, 3, and 5 weeks using a grip strength meter (Columbus Instruments, OH, USA). At the end of the oral administration period, mice were placed with their forelimbs or all limbs on a grid and the grip strength was measured immediately before mice fell from the bar.

### 4.5. Treadmill Exercise Test

Before the treadmill exercise, mice were adapted for 1 week to become familiar with the treadmill (6-lane treadmill, JD-A-09; Jeung Do Bio & Plant Co., Ltd., Seoul, Korea). The exercise consisted of treadmill running at speed of 5 m/min for 10 min, 10 m/min for 10 min, 15 m/min for 10 min, 20 m/min for 10 min, 25 m/min for 10 min, and 25 m/min with no inclination of the treadmill at 1, 3, and 5 weeks. If mice declined to run, then they were motivated by a transient and mild electric stimulation from the treadmill exercise platform. The total distance traveled by each mouse was calculated at the time the mice became exhausted.

### 4.6. Serum Biochemical Analyses

Blood was collected via the abdominal vein at the time of euthanasia. After clotting, plasma was separated by centrifugation at 6000× *g* for 10 min at 4 °C. The lactate, triglyceride, creatinine, blood urea nitrogen (BUN), aspartate aminotransferase (AST), and alanine aminotransferase (ALT) concentrations were measured by commercial ELISA.

### 4.7. Hematoxylin and Eosin Staining

The gastrocnemius muscle in hindlimb samples was fixed with 4% paraformaldehyde at room temperature for 24 h. The tissues were then paraffin embedded, and the resulting blocks were cut into 4 µm sections and stained with hematoxylin and eosin (H&E) to assess the histology. Sectioned tissues were analyzed using a Nikon Eclipse E600 microscope (Nikon Corporation, Tokyo, Japan). The muscle fiber size in hematoxylin and eosin staining data of GA muscle calculated using relative average area of 25–30 muscle fibers were quantified per the same area using Image J software

### 4.8. Quantitative Reverse-Transcription Polymerase Chain Reaction Analysis

RNA was isolated from adipocytes or homogenized tissues using an Easy-Spin total RNA extraction kit (iNtRON Biotechnology, Seongnam, Gyeonggi-do, Korea). cDNA was then obtained from 1 μg RNA on a thermal cycler (Bio-Rad) using a Maxime RT PreMix (iNtRON Biotechnology) for 60 min. The cDNA was analyzed by qRT-PCR (Applied Biosystems, Carlsbad, CA, USA) using the TaqMan probe-based gene expression analysis system in combination with TaqMan gene expression master mix containing ROX dye (Applied Biosystems). The primers of the genes used in the experiment were as follows: myoblast determination protein 1 (MyoD, Mm00440387_m1), myogenic factor 4 (MyoG, Mm00446195_g1), myosin heavy chain 1 (MYH1, Mm01332489_m1), myosin heavy chain type (Myf5, Mm00435125_m1), F-box protein (Atrogin1, Mm00499523_m1), muscle RING-finger protein-1 (MuRF1, Mm01188690_m1), transcription factor A (TFAM1, Mm00447585_m1), nuclear respiratory factor 1 (NRF1, Mm01135609_m1), mitochondrial uncoupling protein (UCP3, Mm01163394_m1), and glyceraldehyde 3-phosphate dehydrogenase (GAPDH, Mm99999915_g1). Expression data were normalized to GAPDH. mRNA levels were calculated as a ratio, using the 2^−ΔΔ*C*T^ method for comparing between groups of data generated by qRT-PCR.

### 4.9. Western Blotting

Muscle tissues were lysed using pro-prep buffer (iNtRON Biotechnology Inc., Seoul, Korea) containing proteinase inhibitors and phosphatase inhibitors. Homogenates were centrifuged at 12,000× *g* for 20 min at 4 °C, supernatants were collected, and the protein concentration was measured using a protein assay kit (Bio-Rad, Hercules, CA, USA). Protein samples (20 µg) were resolved on 8–12% SDS-PAGE gels, and then transferred to PVDF membranes. Primary antibodies against the following proteins were used: SirT1 (D1D7, Cell Signaling Technology, MA, USA), PGC1α (PA5-38021, Invitrogen, Carlsbad, CA, USA), myostatin (PA5-11936, Invitrogen), and glyceraldehyde 3-phosphate dehydrogenase (GAPDH, Cell Signaling Technology). The membranes were blocked with 5% non-fat dried milk for 2 h, and then incubated with secondary antibody conjugated to IgG horse-radish peroxidase.

### 4.10. Cell Culture and Treatment

C2C12 mouse skeletal muscle myoblasts (CRL-1772) were obtained from the American Type Culture Collection (ATCC, Manassas, VA, USA). The cells were maintained in DMEM medium containing 10% Gibco fetal bovine serum (Thermo Fisher Scientific, Massachusetts, MA, USA), 1% antibiotic–antimycotic solution (Thermo Fisher Scientific), and 3.7 g/L sodium bicarbonate in a 5% CO_2_ humidified incubator at 37 °C. To differentiate the myoblasts, 80% confluent myoblasts were incubated in DMEM containing 2% Gibco horse serum (Thermo Fisher Scientific) for 5 days, and the medium was refreshed daily. HY7715 medium was prepared in MRS broth (BD Difco, Sparks, MD, USA) and diluted to a final concentration of 10^6^ CFU/5 × 10^5^ cells. TNFα (100 ng/mL) was used to induce atrophy of C2C12 cell culture.

### 4.11. Cell Viability Test

Cells were seeded (5 × 10^4^ cells/well) in 96-well plates and incubated overnight in growth medium. Cells were then treated with HY7715 culture medium (10^3^, 10^4^, 10^5^, 10^6^, 10^7^, and 10^8^ CFU/well) and incubated for a further 24 h. Next, 0.5 mg/mL 3-(4,5-dimethyl-2-thiazolyl)-2,5-diphenyl-2H-tetrazolium bromide (MTT) solution was added to each well and the cells were incubated for 3 h. The MTT-containing medium was removed, and 150 μL DMSO was added to elute formazan crystals. The absorbances of the eluates were measured at 595 nm on a plate reader (BioTek, Winooski, VT, USA).

### 4.12. Statistical Analysis

The mRNA and protein data are expressed as means and standard deviations (SDs). Data were analyzed by one-way ANOVA, followed by Duncan’s test (IBM SPSS Statistics Version 20.0, Chicago, IL, USA). Values indicated by letters in the figures are significantly different, *p* < 0.05 (a > b > c > d). Tissue masses and microbiome data are expressed as mean ± SD; Student’s *t*-test was used to analyze the data, and values were considered significant when * *p* < 0.05, ** *p* < 0.01, *** *p* < 0.001. All data of the microbiome are expressed as mean ± standard error mean (SEM). Significant differences between groups are presented as ^*^ p < 0.05, ** p < 0.01, and *** p < 0.0001.

## Data Availability

Data are contained within the article.
